# Plant bioindicators of pollution in Sadat City, Western Nile Delta, Egypt

**DOI:** 10.1371/journal.pone.0226315

**Published:** 2020-03-11

**Authors:** Mohamed F. Azzazy

**Affiliations:** Plant Ecology, Surveys of the Natural Resources Department, Environmental Studies and Research Institute, University of Sadat City, Egypt; Gifu University, JAPAN

## Abstract

Anthropogenic pollution can take various forms and affect the air, water, soil, and plants. Monitoring pollution via compounds formed in living organisms such as plants, so-called bioindicators, may be a useful approach for environmental monitoring. The purpose of this study was to investigate and compare plants growing in industrial and residential areas of Sadat City, Egypt, as bioindicators and biomarkers of industrial pollution. Phenolic compounds, flavonoids, and metals were measured in *Bougainvillea glabra* (paperflower) leaves by HPLC-MS, neutron activation analysis, and atomic absorption spectrophotometry. Air, water, and soil samples associated with *B*. *glabra* sampled in industrial and residential areas were also analyzed for the presence of phenolic compounds, flavonoids, metals, and particulate matter. There were significantly higher levels of flavonoids and phenolic compounds in the leaf extracts of plants growing in industrial areas compared to those growing in residential zones (P<0.05). Metal accumulation in leaves was also significantly higher in the industrial zone than the residential zone: iron, lead, zinc, nickel, and manganese were present at significantly higher levels in plants in the industrial zone compared to those growing in the residential zone (P<0.05); nevertheless, the concentrations of heavy metals in the air, water, and soil were under local legal environmental limits. This study demonstrates that pollution has significant effects on total phenolic, flavonoid and metal levels in *B*. *glabra* plants, not only demonstrating the effects of pollution on ecosystem health but also paving the way for using plants as bioindicators and for phytoremediation.

## Introduction

Air- and water-borne pollution is an undesirable consequence of industrialization that is having a growing impact on productivity, health, and climate change [1–5]. Pollution affects plants and animals via a number of routes including through pollutants dissolved in the rain (e.g., sulfur dioxide producing sulfuric acid), chemical discharge into water courses, and particulate matter (PM) in the air including dust, dirt, and smoke. PM and gases such as nitrogen monoxide (NO), nitrogen dioxide (NO_2_), carbon monoxide (CO), and sulfur dioxide (SO_2_) are traffic-related gaseous pollutants [6], levels of which are associated with and altered by meteorological variables such as wind speed, wind direction, temperature, and humidity [7–9]. Silicate minerals and anthropogenic metal-bearing particles have now been described as minor abundant phases in snow deposits in the vicinity of power plants [10]. Silver, sodium, and zinc have been detected at high concentrations in black poplar leaves [11]. Anthropogenic pollution is thus extending to every corner of the ecosystem and planet, including plants. Monitoring and assessing pollution in plants and in the air, water, and soil from industrial sources may therefore be an important way to detect and manage the impact of industry and pollution on our environment [4].

Bioindicators are living organisms such as plants, planktons, animals, and microbes with the capacity to monitor the health of the environment. They are therefore a useful means to measure the negative impacts of industrial activity on the environment [12]. Similarly, environmental biomarkers are identifiable measures (e.g., chemicals or genes) of environmental processes [13] and a useful tool not only to monitor and evaluate the environmental state but also develop our knowledge of molecular toxicity mechanisms in different animal and plant species in the ecosystem [14, 15]. Therefore, plants can be used to assess whether certain ecophysiological responses may be useful biomarkers of urban pollution. Some plant biomarkers are specific to only one pollutant or group of pollutants, while others respond to a wide range of pollutants and/or stressors [16]. For example, flavonoids and phenolic compounds indicate stress in plants [17, 18]. These secondary metabolites, in particular polyphenols, are of particular importance in plant-environment relationships [19]. High concentrations of metals in plants can also inhibit chlorophyll production, increase oxidative stress, and weaken stomata resistance [20].

*Bougainvillea glabra* (family: Nyctaginaceae) is a common ornamental plant grown in tropical and subtropical gardens that is often grown as a shrub or climber [21]. The aim of the present study was to investigate using *B*. *glabra* as a bioindicator and assess the accumulation of phenolics, flavonoids, and metals in their leaves as biomarkers of environmental pollution.

## Materials and methods

### Study area

**Fig 1:**
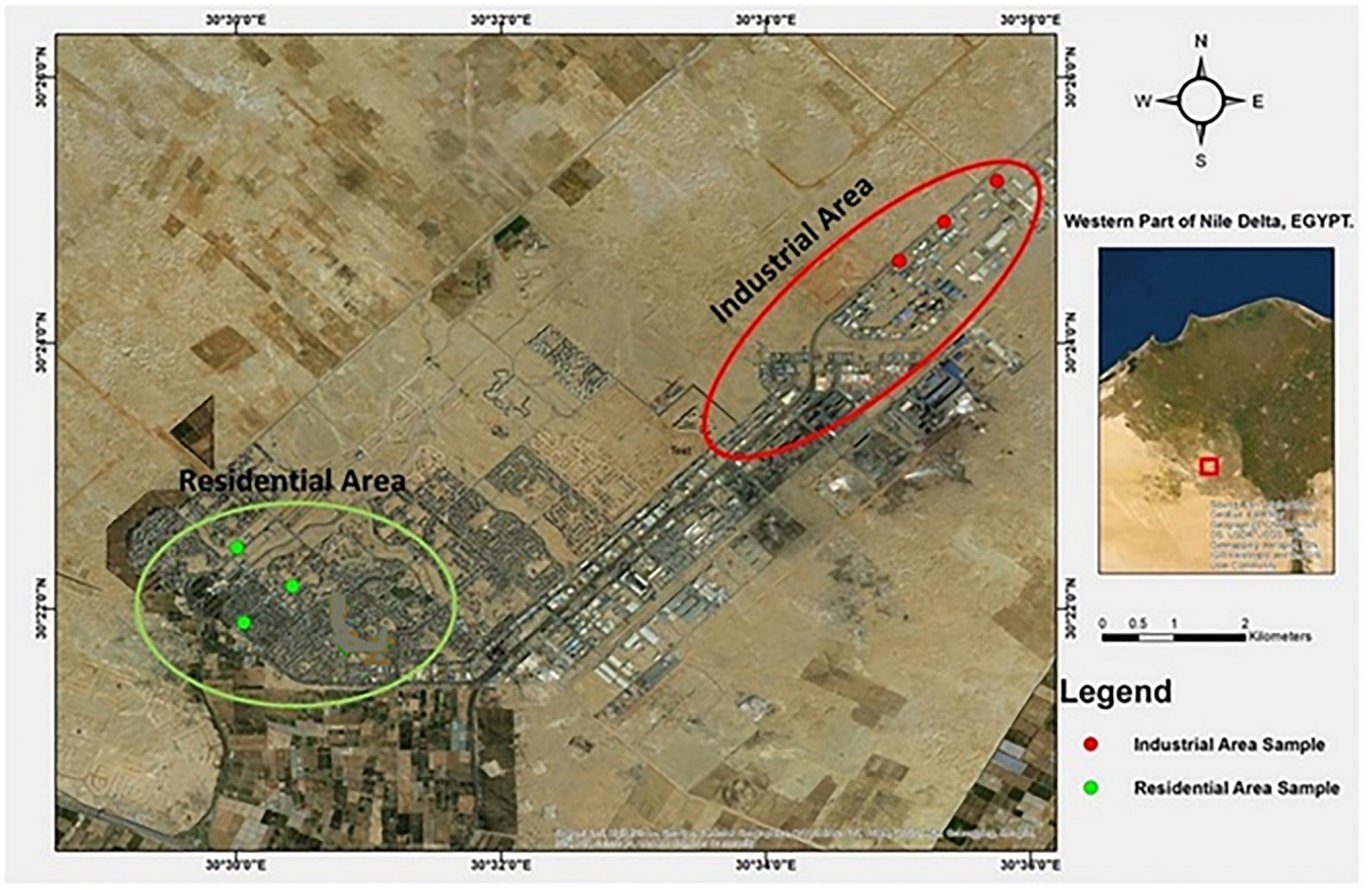
An Environmental Studies and Research Institute (ESRI) image showing the sites of sample collection in the study area.

Sadat City is located about 100 km north of Cairo, Egypt. The city is bounded 30°19’30”-30°40’27” E longitude and 30°15’50”-30°34’00”N latitude (Fig 1), from the east by Kafer Dawoud and El Khatataba, from the west by El Birigat, and from the north by Nubariya Canal and El Tahrir. The industrial zones are located in a separate spine along the south-eastern edge of the city to ensure that industrial pollution travels downwind. To protect the city from wind and storms, a green shelterbelt of trees about 35,000 feddans (1 feddan = ~175 m^2^) was planted around the city containing about 2,000 feddans of vegetables and fruit.

The residential districts and industrial zones of Sadat City cover about 50 km^2^ of the study area. With a future planned capacity of 750,000 inhabitants, the city currently hosts a population of about 200,000 inhabitants and about 100,000 seasonal residents working in industry and agriculture. The city has one waste disposal site at the southern side of the city behind the industrial zone about 700 feddans in area.

### Sampling

The study area was sampled at the sites shown in Fig 1 in March and April 2018. Three sites were sampled in the residential zone and three in the industrial zone, with plant and soil samples taken at each. PM_10_ (particulate matter <10 micrometers) and heavy metal samples were collected using dust fall collectors placed on the roofs of buildings (12 m tall and 30–40 m from the sampling sites according to [22]) in the two sampling zones, and water samples were taken from wells present at the same sites. Plants were studied morphologically; flavonoids and phenolic compounds were investigated in *B*. *glabra* leaves by HPLC-MS, while metals were measured in leaves using neutron activation analysis and atomic absorption spectrophotometry. The Environmental Studies and Research Institute (ESRI), University of Sadat City Egypt, has permission to make regular environmental investigations to the industrial zone for Environmental Impact Assessments (EIA), and therefore no new permits were required for this study.

### Climatic parameters

The Tropical Rain Measurement Mission (TRMM) records three hourly rainfall measurements and is freely available online including from the TOVAS website (https://serc.carleton.edu/resources/22961.html). Climate was also estimated using Egyptian Meteorological Authority data between January and December 2018.

### Identification of the collected plants in the study area

Plants in industrial and residential areas were identified using well identified herbarium specimens of the Environmental Studies and Research Institute (ESRI) and the Herbarium Faculty of Science Botany Department, Mansoura University. A voucher specimen was deposited at the herbarium of the ESRI, University of Sadat City. The morphological features of plants growing in both locations were compared.

### Biomarker analysis

HPLC-MS Ultimate 3000/Amazon SL ion trap mass spectrometry was undertaken using Acclaim 2.2 μm 120 A 2.1–150 mm columns (Thermo Fisher Scientific, Waltham, MA). The HPLC-MS device was used to detect flavonoids and phenolic compounds in plant leaves.

### Chemicals and reagents

Methanol, acetonitrile, and deionized water were of HPLC-MS grade and were from Sigma Aldrich (St Louis, MO).

### Plant materials and sample preparation

Leaves were collected from plants growing in the study areas. 50 g of leaf samples were taken from *B*. *glabra* plants of the same age, uniformly from the lower foliage. Care was taken to avoid selecting leaves with insect infestations, honeydew, or bird droppings. Samples were preserved in clean paper envelopes before being taken to the laboratory for air drying at room temperature for 48 h and then being crushed to a fine powder with a pestle and mortar. Methanol extraction was performed with a Soxhlet apparatus [23], enhancing extraction efficiency by the application of the methods described in [24, 25]. For HPLC-MS analysis, 0.1 g of powdered leaves was mixed with 10 ml 70% methanol and placed on a rotating shaker at 200 rpm at 40°C for 16 h. The filtrate was collected and filtered through a 0.45 μm nylon filter. 20 μL of plant extract was injected into the HPLC-MS column. All sample solutions were stored at 4°C prior to use. The compound at each peak was identified according to its molecular weight and retention time.

### Determination of phenolic contents

The total phenolic contents of plant leaf extracts were determined using the Folin–Ciocalteu method [26]. Briefly, 1 mL of extract (100–500 g/ml) solution was mixed with 2.5 ml of 10% (w/v) Folin–Ciocalteu reagent. After 5 min, 2.0 ml of Na_2_CO_3_ (75%) was added to the mixture and the sample incubated at 50°C for 10 min with intermittent agitation. Then, the sample was cooled and the absorbance measured using a UV spectrophotometer (Shimazu, UV-1800) at 765 nm against a blank without extract. Data were expressed as mg/g of gallic acid equivalents in milligrams per gram (mg GAE/g) of dry extract.

### Determination of flavonoid contents

The flavonoid contents of plant leaf extracts were measured as per the Dowd method [27]. An aliquot of 1 ml of extract solution (25–200 g/ml) or quercetin (25–200 g/ml) was mixed with 0.2 ml of 10% (w/v) AlCl_3_ solution in methanol, 0.2 ml (1 M) potassium acetate, and 5.6 ml distilled water. The mixture was incubated for 30 min at room temperature followed by the measurement of absorbance at 415 nm against the blank. The outcome data were expressed as mg/g of quercetin equivalents in milligrams per gram (mg QE/g) of dry extract.

### Instrumentation

Plant specimens were studied for flavonoids and phenolics in the central laboratory of the ESRI, University of Sadat City, Egypt. The binary mobile phase consisted of solvents A (1% formic acid) and B (acetonitrile with 1% formic acid). A mobile phase gradient was used as follows: 0 min, A: B 10–90; 36 min, A: B 70–30; 50 min, A: B 100–0. After each run, the chromatographic system was equilibrated with 20% B for 30 min. The injection volume was 20 μl and the flow rate was 0.8 ml/min. The effluent was split 2:3 using a micro-splitter valve before introduction into the mass spectrometer. UV traces were measured at 290, 254, and 350 nm and UV spectra (DAD) were recorded between 190 and 900 nm according to [28].

### Gas analysis

NO, NO_2_, and NOx were measured with the Thermo Environmental Instruments 42C NO, NO_2_, NOx Analyzer, EPA reference method RFNA-1289-074, over 10–300 seconds. SO_2_ was measured with the Thermo Environmental Instruments 43C SO_2_ Analyzer, EPA equivalent method EQSA-0486-060, over 10–300 seconds. CO was measured with the Thermo Environmental Instruments 48C CO Analyzer, EPA reference method RFCA-0981-054, over 10–300 seconds. Outdoor NO_2_ concentrations were measured using the method in [29]. Gases were measured in the work environments using a Testo Portable Emission Gas Analyzer (Testo Inc., Sparta, NJ; Model 350M/XL).

### Particulate matter (PM)

Sampling was conducted in March and April 2018. PM was collected at the study sites using the filtration method [30], which depends on sample aspiration through a cellulose ester membrane filter (0.45 μm pore size) using a vacuum pump at a flow rate of 17 L/m [31] to trap particles less than 0.1 μm in diameter for chemical analysis [32, 33]. A total suspended particle (TSP) meter (Kanomax, Andover, NJ) was used to measure TSPs in the work environment and a PM_10_ apparatus was used to measured PM_10_ [31].

Deposited particulate matter was collected monthly for two months using dust fall collectors placed on the roofs of buildings (12 m tall) in the two study zones (industrial and residential) [30]. Filters were digested with 6 ml nitric acid, 2 ml hydrogen peroxide, and 0.1–1 ml hydrofluoric acid at 180°C for 6 h. Then, the solution was passed through a filter (pore size 0.45 μm; Membrana Wuppertal).

### Atomic absorption spectrophotometry

Atomic absorption spectrophotometry was performed using a Spectrum 100 (Perkin Elmer, Waltham, MA) to determine heavy metal concentrations in plants.

### Neutron activation analysis

The concentration of sodium (Na), calcium (Ca), magnesium (Mg), chloride (Cl), and manganese (Mn) in plant tissues was assessed by neutron activation analysis (NAA) using the IBR-2 Reactor (IBR2 of the Joint Institute for Nuclear Research, Dubna, Russia).

### Groundwater samples

Two water samples were collected from two wells in the study areas (industrial and residential, one sample from each). The water samples were acidified and stored in an icebox before being analyzed by atomic absorption spectroscopy (AAS) at the Water Department, Central Health Laboratories, Ministry of Health and Population, Egypt.

### Soil chemical analysis

Soil samples were collected from the industrial and residential zones (three samples from each at the plant sampling sites), mixed, and crushed with a with an iron-free grinder to avoid contamination of samples with iron. One gram of each sample was placed in a Teflon beaker and digested with 10 ml HF and 2 ml HClO_4_ before being heated to nearly dry the sample. The residual material was dissolved with HCl (12 N) and the filter diluted with 25 ml deionized water [34, 35]. Chemical analysis was performed by atomic absorption spectroscopy (AAS) for chromium (Cr), nickel (Ni), and lead (Pb) [34].

### Statistical analysis

Data are expressed as mean ± standard deviation (SD). Data were analyzed with t-tests using SPSS ver. 14.0 (IBM Statistics, Chicago, IL). A P-value < 0.05 was considered statistically significant.

## Results

### Climatic study

Monthly mean temperatures over the study period varied from a minimum of 12.4°C in January to a maximum of 36.4°C in July over the study period (Table 1). Rainfall ranged from a total of 21.4 mm in January to no rainfall in June, July, and August. Average relative humidity ranged from 45.0% in December to 71.0% in August. Average wind speed varied from 6.475 m/s in October to 10.36 m/s in March.

**Table 1:** Monthly means of meteorological parameters from January to December 2018.

Month	Air temperature (°C)	Rainfall (mm/month)	Relative humidity (RH) %	Wind speed (km/h)
January	12.4	21.4	56.0	9.435
February	13.5	15.2	57.0	9.99
March	15.7	16.7	60.0	10.36
April	19.3	16.8	62.0	9.99
May	22.7	15.9	66.0	7.955
June	25.8	Trace	68.0	8.325
July	36.4	Trace	70.0	7.215
August	37.8	Trace	71.0	6.66
September	25.0	2.6	70.0	7.955
October	22.1	7.1	67.0	6.475
November	17.8	18.3	55.0	7.03
December	13.8	16.0	45.0	7.77

### Morphological changes in plant leaves due to pollution

**Fig 2:**
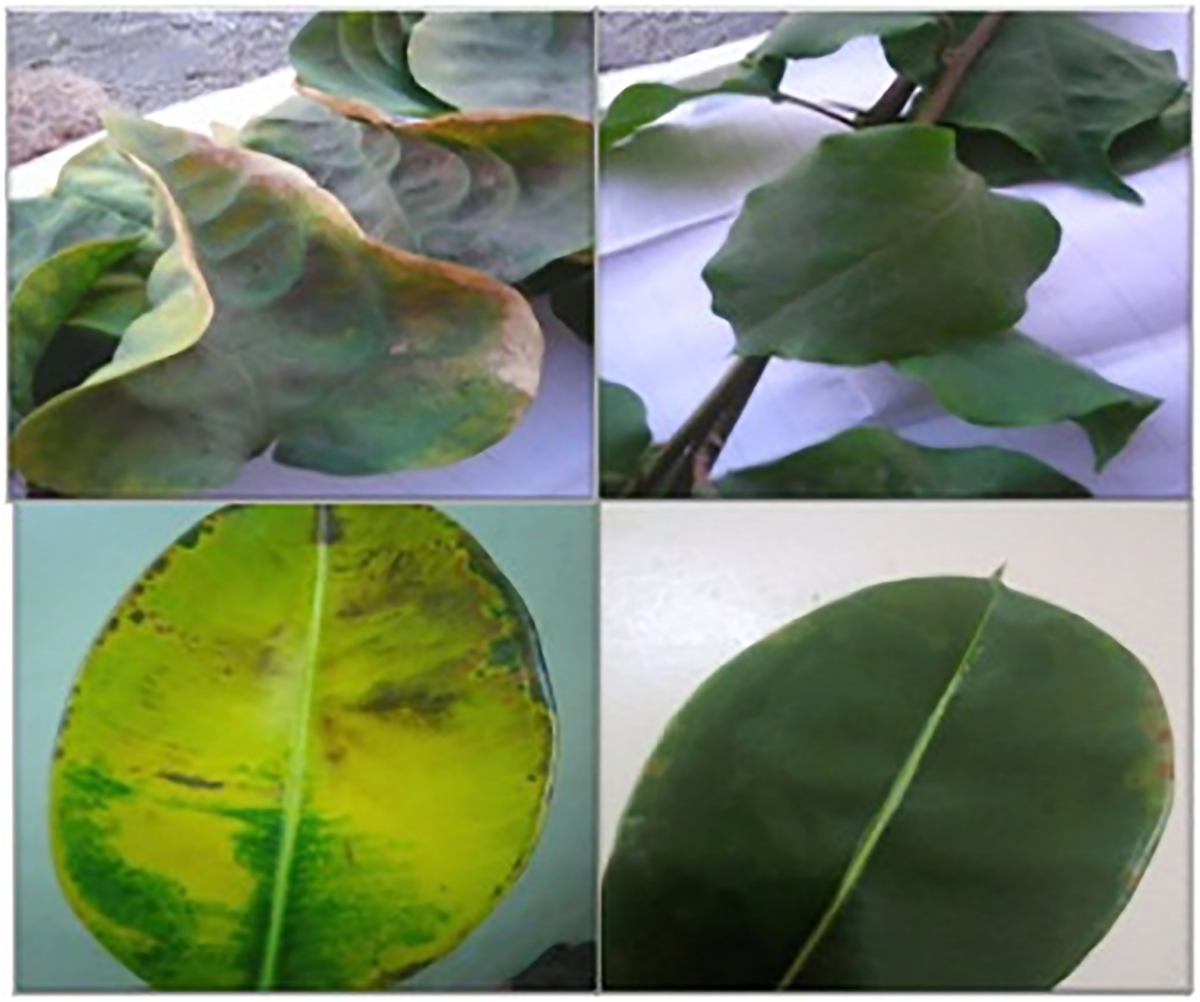
Illustrative morphological differences in *Bougainvillea glabra* growing in industrial (left) and residential (right) zones.

Overall, the leaves of plants in the industrial zone had discolored, dusty, and wrinkled leaves compared to their counterparts growing in the residential zone, which had smooth surfaces and edges and were clean with no wrinkles and normal edges (Fig 2).

### Metal concentrations in plants growing in industrial and residential zones

Table 2 shows the average metal concentrations in *Bougainvillea glabra* leaves growing in the industrial and residential zones. Iron (494.00 ± 1.63 vs. 0 ppm), zinc (445.00 ± 2.41 vs. 33.28 ± 2.41ppm), lead (0.0066 ± 0.001 vs. 0 ppm), nickel (74.11 ± 0.10 vs. 53. 31 ± 1.7 ppm), and manganese (452.47 ± 3.9 vs. 211.00 ± 6.3 ppm) were all present at significantly higher levels in plants growing in the industrial zone than the residential zone (P<0.05), while copper and cadmium were undetectable in both.

**Table 2:** Heavy metal content in *Bougainvillea glabra* leaves in the industrial and residential zones.

Item	Industrial Zone	Residential Zone	Sign.
Fe (ppm)	494.00 ± 1.63	0.0 ± 1.63	P<0.05
Zn (ppm)	445.00 ± 2.41	33.28 ± 2.41	P<0.05
Pb (ppm)	0.0066 ± 0.001	0.0 ± 0.001	P<0.05
Ni (ppm)	74.11 ± 0.10	53.31 ± 1.7	P<0.05
Mn (ppm)	452.47 ± 3.9	211.00 ± 6.3	P<0.05
Cu (ppm)	0.00	0.00	NS
Cd (ppm)	0.00	0.00	NS

**Abbreviations**: NS = not significant, ppm = parts per million; values are means ± SD from three replicates.

### Plant flavonoid analysis

**Fig 3:**
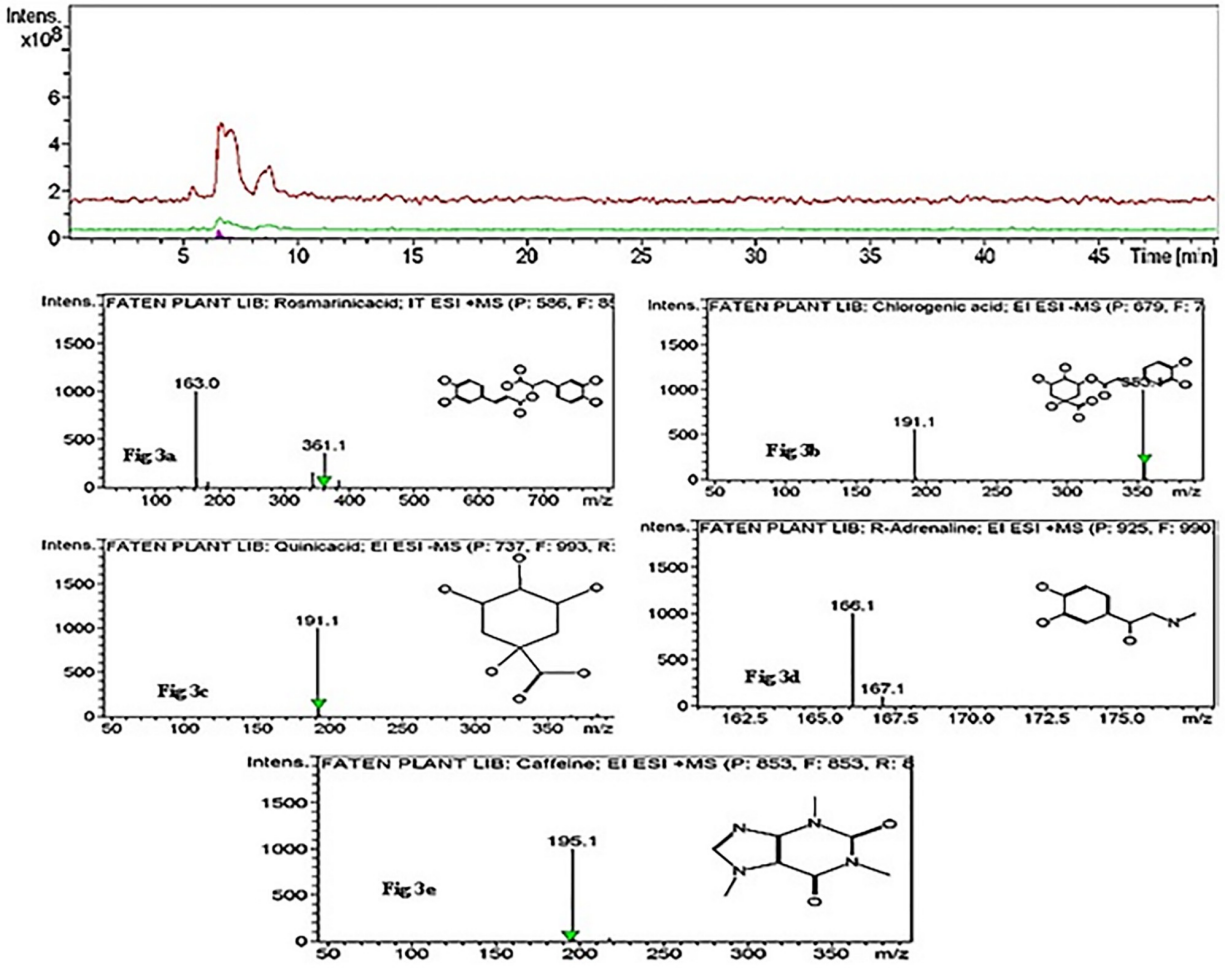
HPLC-MS analysis of methanolic extracts of *Bougainvillea glabra* leaves growing in the industrial zone.

HPLC-MS analysis of *B*. *glabra* (Table 3 and Fig 3) leaf extracts revealed a set of peaks in plant extracts derived from the industrial zone. Twenty-one compounds were detected in industrial zone leaves; five major peaks were selected on the basis of peaks and retention times (Rt). Rosmarinic acid had an Rt of 48.9 min and peak area percentage of 12.01%; chlorogenic acid had an Rt of 6.7 min and peak area of 3.9%, quinic acid had an Rt of 32.0 min and peak area of 3.8%, R-adrenaline had an Rt of 11.0 min and peak area of 2.9%, while caffeine had an Rt of 16.8 min and peak area of 2.5%.

**Table 3:** HPLC-MS analysis of methanolic extracts of *Bougainvillea glabra* leaves growing in the industrial and residential zones.

Industrial Zone								
#	Rt (min)	Area	Chemical Formula	Compound Name	Intensity	Range (min)	Area %	Int. %
264	3.0	128922.3	C15H22N2O4	(L) Tyrosyl-L-leucine	10091	2.8–3.1	0.1	0
472	5.5	6944	C11H21NO4	Isobutyryl carnitine	2303	5.0–5.9	0.6	0
498	5.6	173466.7	C6H6O3	Phloroglucinol	36603	5.6–5.7	0.2	1
598	6.6	2520797	C10H19NO4	BOC valine	115237	6.3–7.1	2.2	19
603	6.7	25	C16H18O9	Chlorogenic acid	35	6.5–6.8	3.9	0.4
638	6.9	27714	C6H10O3	2-Oxohexanoic acid	101243	6.7–7.5	2.5	2
3770	46.3	280901.1	C18H39NO3	Phytosphingosine	10576	45.9–46.5	0.3	0
3990	48.9	32388.1	C18H16O8	Rosmarinic acid	5120	48.8–49.0	12.01	10
665	7.1	21737	C8H10O2	4-Hydroxyphenylethanol	13513	7.0–7.3	2.0	2
682	7.5	584315.0	C17H33NO4	Decanoyl-L-carnitine	20242	7.3–7.8	0.5	0
1114	17.0	1751314.7	C15H10O4	Daidzein	60860	16.8–17.2	0.4	0
1102	16.8	2302295	C8H10N4O2	Caffeine	5761	17.1	2.5	3.2
1713	20.8	111325.2	C13H25NO4	Hexanoyl-L-carnitine	15612	22.5–22.8	0.1	2
1683	20.5	299060.8	C5H12O5	Ribitol	46576	20.2–20.7	0.3	1
1963	24.2	136154.4	C6H5NO2	Isonicotinicacid	13267	24.1–24.3	0.1	0
925	11.0	1189877.7	C9H13NO3	R-Adrenaline	58886	10.7–11.2	2.9	2
2426	29.9	148877.8	C10H15NO	Hordenine	11543	29.7–30.8	0.1	0
2533	24.8	1386306.8	C15H10O5	Genistein	11352	24.5–24.9	1.3	2
2635	32.3	641082	C12H23NO4	Isovalerylcarnitine	45205	32.2–32.5	0.6	1
2757	33.8	82761.4	C21H24O10	Phlorizin	7046	33.7–33.9	0.1	0
2286	32.0	1366521.9	C7H12O6	Quinic acid	71701	31.6–32.3	3.8	1
**Residential Zone**								
240	2.5	1017309.7	C21H24O10	Phlorizin	59274	6.4–7.3	1.9	2
659	7.3	2659727.5	C10H13NO	Phenacetin	125701	7.2–7.5	1.0	2
669	7.4	165148.5	C16H18O9	Chlorogenic acid	22810	12.2–12.7	0.3	1
1041	12.5	1424751.3	C28H34O15	Hesperidin	96603	14.0–14.4	3.7	3
1157	14.2	543080.9	C5H12NO	Betaine aldehyde	36220	16.3–16.8	1.0	1
1349	16.6	48288.9	C6H7N5	6-Methyladenine	3681	17.1–17.6	0.1	0
3286	42.4	1119499.3	C13H25NO4	Hexanoyl-L-carnitine	67359	41.6–41.9	0.8	2
3293	41.7	3293 41.7	C8H8O4	2-hydroxy-5-methoxybenzoic	6503	42.4–42.7	0.1	2
3301	42.6	2181244.8	C18H16O8	Rosmarinic acid	100251	48.6–48.9	1.1	1
3819	48.7	114496.2	C15H10O5	Genistein	10172	48.6–49.1	2.2	2
3892	48.9	656974.6	C15H10O4	Daidzein	45629	48.6–49.1	2.5	2
1683	20.5	299060.8	C5H12O5	Ribitol	46576	20.2–20.7	0.2	1
1713	20.8	111325.2	C13H25NO4	Hexanoyl-L-carnitine	15612	22.5–22.8	0.2	2
598	6.6	2520797	C10H19NO4	BOC Valine	115237	6.3–7.1	1.3	1
2635	32.3	641082	C12H23NO4	Isovalerylcarnitine	45205	32.2–32.5	0.5	1
498	5.6	173466.7	C6H6O3	Phloroglucinol	36603	5.6–5.7	0.2	1
3770	46.3	280901.1	C18H39NO3	Phytosphingosine	10576	45.9–46.5	0.4	0

**Fig 4:**
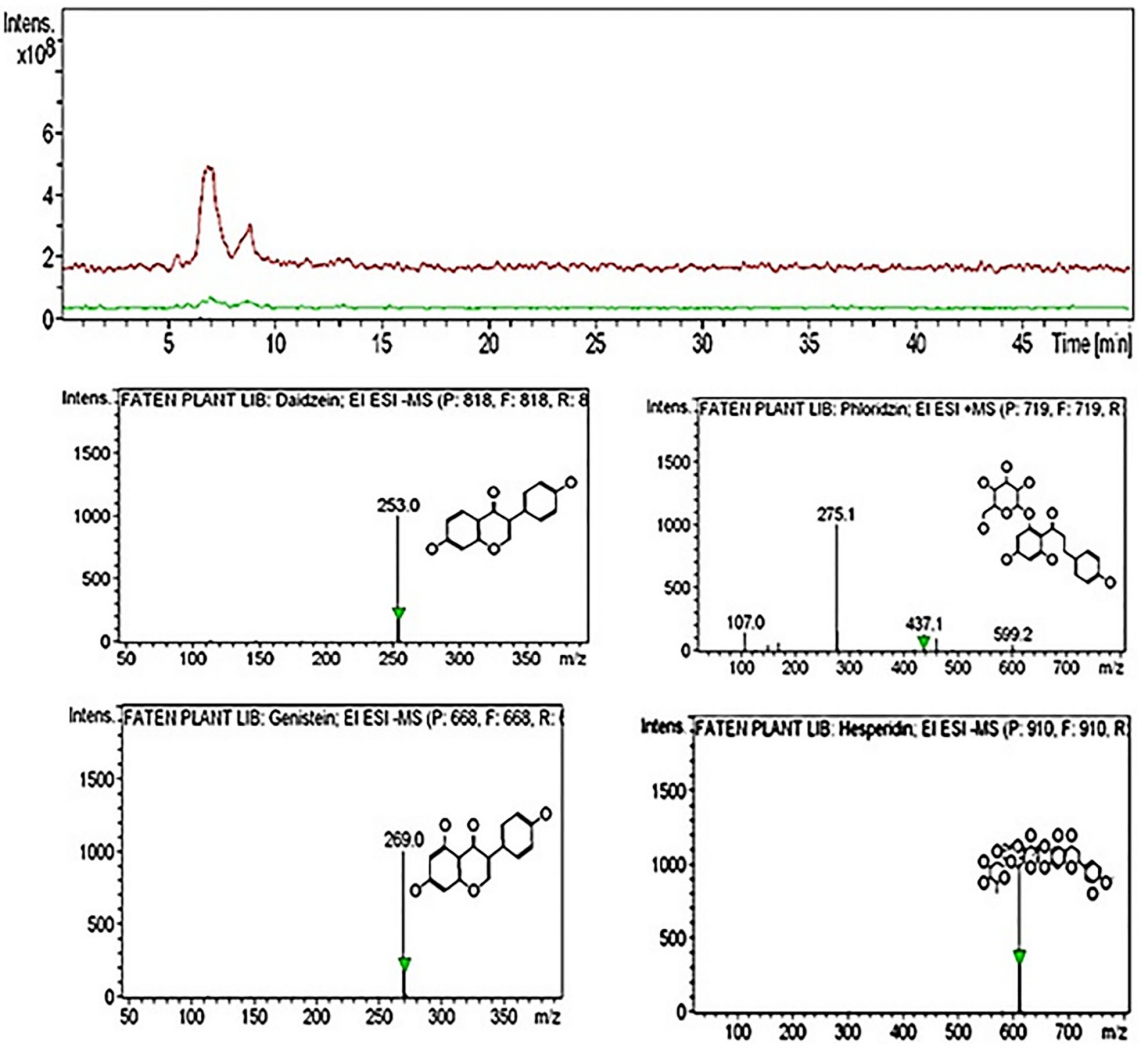
HPLC-MS analysis of methanolic extracts of *Bougainvillea glabra* leaves growing in the residential zone.

The HPLC-MS analysis of *Bougainvillea glabra* from the residential area (Table 3, Fig 4) revealed seventeen compounds all identifiable as flavonoids, four of which were major peaks with retention times of 5 min and over: hesperidin (Rt 12.5 min), peak area percentage 3.7%; daidzein (Rt 48.9 min), peak area 2.5%; genistein (Rt 48.7 min), peak area 2.2%, and phlorizin (Rt 2.5 min), peak area 1.9%.

Taken together, the total phenolics and flavonoids of *B*. *glabra* were significantly higher in the industrial zone than in the residential zone (P<0.05; Table 4).

**Table 4:** Determination of total phenolic and flavonoid contents in *Bougainvillea glabra* leaf extracts. Values are means ± SD from three replicates.

	Industrial Zone	Residential Zone	Sig.
**Total phenolics**	61.72 ± 0.70 GAE mg/100g	45.82 ± 0.50 GAE mg/100g	P<0.05
**Total flavonoids**	233.53 ± 16.10 QE mg/100g	212.23 ± 9.05 QE mg/100g	P<0.05

**Abbreviations**: GAE = gallic acid equivalents; QE = quercetin equivalents; Sig. = significance

### Total suspended particulates (TSP, PM_10_) and gases in the industrial and residential zones

Table 5 shows that there were significantly higher concentrations of TSPs, PM_10_, and gases at the industrial zone compared to the residential zone.

**Table 5:** Means of TSP-PM_10_ and gases in the industrial and residential zones during March and April 2018 (mg/m³). Values are means ± SD from three replicates.

Item	Study Zone and Value		Sig.
TSP	Industrial	7.06 ± 2.3	P<0.05
	Residential	0.40 ± 0	
PM_10_	Industrial	15.72 ± 6.5	P<0.05
	Residential	0.0 ± 0.0	
NO	Industrial	9.66 ± 5.0	P<0.05
	Residential	0.0 ± 0.0	
NO_2_	Industrial	0.12 ± 0.04	P<0.05
	Residential	0.04 ± 0.0	
NOx	Industrial	68.43 ± 1.66	P<0.05
	Residential	0.0 ± 0.0	
SO_2_	Industrial	16.76 ± 12.74	P<0.05
	Residential	0.05 ± 0.0	
CO	Industrial	42.16 ± 24.1	P<0.05
	Residential	0.0 ± 0.0	
CO_2_	Industrial	473.00 ± 35.5	P<0.05
	Residential	0.01 ± 0.0	

**Abbreviations**: PM = particulate matter; Sig. = significance; TSP = total suspended particles

### Groundwater analysis

Table 6 shows that turbidity, total distilled solids (TDS), pH, chlorides, fluorides, sulfates, hardness, calcium, magnesium, silicon dioxide, zinc, and selenium were all present at significantly higher levels in water from the industrial zone than the residential zone (all P<0.05). Potassium and barium were significantly lower in water from the industrial zone than the residential zone (all P<0.05).

**Table 6:** Water analysis from wells in the residential and industrial zones. Values are means ± SD from three replicates.

Parameters	Industrial zone well	Residential zone well	Sign.
Turbidity (NTU)	0.3500 ± 0.15000	0.20000 ± 0.00	P<0.05
TDS (ppm)	426.500 ± 1.5000	248.000 ± 0.00	P<0.05
pH	8.100 ± 0.1000	7.6000 ± 0.00	P<0.05
Ammonia (ppm)	ND	ND	NS
Nitrite (ppm)	ND	ND	NS
Nitrate (ppm)	ND	ND	NS
Chlorides (ppm)	93.000 ±1.0000	20.000 ±1.000	P<0.05
Fluorides (ppm)	0.11500 ± 0.0050	0.01400 ± 0.0050	P<0.05
Sulfates (ppm)	39.000 ± 1.0000	17.000 ± 0.70	P<0.05
Total hardness AS CaCO_3_	173.750 ± 0.25000	121.000 ± 0.00	P<0.05
Temporary hardness AS CaCO_3_	170.000 ± 1.0000	119.000 ± 0.00	P<0.05
Permanent hardness AS CaCO_3_	2.4000 ± 0.1000	0.000	P<0.05
Ca hardness AS CaCO_3_	93.000 ± 0.5000	69.000 ± 0.00	P<0.05
Mg hardness AS CO_3_	81.000 ± 1.000	49.000 ± 0.00	P<0.05
Na (ppm)	75.500 ± 0.500	41.000 ± 0.00	P<0.05
K (ppm)	1.500 ± 0.500	2.000 ± 0.00	P<0.05
Ca (ppm)	38.200 ± 0.115	27.000 ± 0.577	P<0.05
Mg (ppm)	20.166 ± 0.611	12.000 ± 0.577	P<0.05
Fe (ppm)	ND	ND	NS
Mn (ppm)	ND	ND	NS
SiO_2_ (ppm)	29.000 ± 0.577	18.000 ± 0.577	P<0.05
Cd (ppm)	ND	ND	NS
Cr (ppm)	ND	ND	NS
Cu (ppm)	ND	ND	NS
Ba (ppm)	0.006 ± 0.00005	0.013 ± 0.003	P<0.05
Pb (ppm)	ND	ND	NS
Zn (ppm)	0.00690 ± 0.00005	0.00343 ± 0.00014	P<0.05
Se (ppm)	0.00113 ± 0.000088	ND	NS

**Abbreviations**: ND = not detected; NTU = nephelometric turbidity unit; NS = not significant; ppm = parts per million; TDS = total distilled solids.

### Chemical analysis of soil samples

Heavy metal concentrations in the soil samples from industrial and residential zones are listed in Table 7. All metals apart from cadmium, which was not detectable, were present at significantly higher levels in the samples obtained from the industrial area than the residential area (all P<0.05).

**Table 7:** Means of heavy metal concentrations in soil samples (ppm) collected from industrial and residential zones. Values are means ± SD from three replicates.

Metal	Industrial Zone	Residential Zone	Significance
Fe	8750.00 ± 21.6	5559.0 ± 0.57	P<0.05
Cu	2.08 ± 0.20	1.37 ± 0.008	P<0.05
Cd	0	0	NS
Zn	12.50 ± 0.0	0.28 ± 0.005	P<0.05
Mn	33.33 ± 4.16	19.71 ± 0.005	P<0.05
Pb	4.57 ±0.15	0.48 ± 0.005	P<0.05
Ni	20.48±2.02	0.500 ± 0.005	P<0.05
Cr	30.29 ± 2.2	23.50 ± 0.005	P<0.05
Co	1.25 ± 0.005	0.39 ± 0.013	P<0.05

## Discussion

Sadat City, located north of Cairo, is an area characterized by extreme aridity, a long hot summer, and a short warm winter, which greatly influences the hydrological properties of the drainage basins [36, 37]. Indeed, over the year in which this study was conducted, mean monthly air temperature ranged from 12.4°C to 37.8°C; the annual mean wind velocity was 6.66 k/h; the mean relative humidity ranged from 45–71%, and rainfall ranged from 2.6–21.4 mm per month. Since polluting gases and PM are altered by meteorological variables [8], in this study sampling was conducted over a relatively short two-month period. The findings show that *B*. *glabra* leaves in the industrial zone in general contained higher levels of phenolics, flavonoids, and metals than those in residential zones, with increased PM and metals present in the air and soil of the industrial zone compared to the residential zone.

The effect of rapid industrialization on human health is a major public health concern [38]. Plants growing in industrial and residential zones revealed marked differences in their leaf morphology. Mahfoozi et al. [39] reported that environmental stresses negatively influence plant growth and productivity and trigger a series of morphological, physiological, biochemical, and molecular changes in plants. Dust deposition on leaf surfaces may also reduce chlorophyll synthesis due to a shading effect [40]. The resulting changes, either morphological or physiological, can be used as bioindicators of the environmental state [41]. Indeed, biomonitoring of air quality using plants is not a new concept and has been applied to detect and to monitor the effects of pollution [42]; here we exploit this concept and show that not only does plant growth in an industrial area alter leaf morphology but also biochemical indicators of stress such as plant flavonoid and phenolic content.

Flavonoids function as stress indicators and accumulate at high levels in many plant tissues in response to a wide range of biotic and abiotic signals [2, 43–45], enhancing the scavenging of free radicals [46]. Flavonoids have also been reported to be a good indicator of environmental contamination, especially of O_3_ pollution [47]. Flavonoids comprise a large and common group of plant phenolics, with more than 5000 different described flavonoids in six major subclasses [48]. Plants may alter their secondary metabolite synthesis, production, secretion, and storage when subjected to the abiotic stress factors [49]. The results obtained in the present study revealed that pollution in the industrial area was associated with elevations in total phenolics and flavonoids compared with samples of plants from non-polluted, residential sites. The elevations in total phenolics and flavonoids may have acted as a stress defense mechanism in plants against these environmental pollutants.

HPLC-MS profiles of the studied plant samples from the industrial zone revealed the presence of 21 compounds, five of which were present as major peaks belonging to flavonoid and phenolic compounds. R-adrenaline was detected in plants growing in the industrial zone, which may due to environmental stress; indeed, Hughes and Wilson [50] reported that adrenaline is known to protect against oxidation by flavonoids, and Cetinkaya et al. [49] reported that flavonoids increase in response to unfavorable conditions. Furthermore, Cannac et al. [51] reported that synthesis of total phenolic compounds increased in *Pinus laricio* over three months, such that total phenolic compounds could be used as bioindicators of short-term responses of pine needles to prescribed burning. By contrast, residential zone plant extracts showed the presence of 17 compounds, four of which were major flavonoid and phenolic peaks. Therefore, total phenolics and flavonoids highly represented in plants in the industrial zone may be a result of stress-inducing pollution [52]. Our results are also consistent with [53], who observed a higher quercetin (phenolic flavonoid) content in samples from a polluted site.

The total concentrations of TSPs in water in the industrial zone were higher than the US Environmental Protection Agency’s prescribed standards for air quality, presumably as a result of emissions from local industrial activity such as steel factories in this area. Generally, however, the outlet values of TSPs in the samples were less than the local legally permitted value (EEAA (Egyptian Ministry for the Environment) Law 4/1994; 9/2009), which is 10 mg/m³ in the work environment. Similarly, PM_10_ levels were higher in the industrial zone and only present at trace levels in the residential zone, but the outlet PM_10_ values in all samples were generally lower than required by law (4/1994; 9/2009). Whilst this is encouraging, it is worth noting that concentrations may have been diluted by the relatively high rainfall during the sampling period (March and April) [54]. Likewise, the relative humidity of 60% and 62% during March and April, respectively, may have affected PM_10_ levels, since when the relative humidity is over 55%, then PM_10_ concentrations are reduced [55].

The higher SO_2_ values at the industrial site may have also affected the plants; NOx dissolves in cells to produce nitrite ions (NO_2_), which is toxic at high concentrations, and nitrate ions (NO_3_) that enter into nitrogen metabolism as if they had been absorbed through the roots. It has been reported that exposure to pollutant gases, particularly SO_2_, causes stomata closure, which protects the leaf against the further entry of the pollutant but also curtails photosynthesis [1]. Nanos and Ilias [56] reported that heavy metal toxicity associated with cement dust contamination can also cause photo system damage. In addition, the presence of dust between the peltate on the lower leaf surface might have caused a decrease in leaf conductance to water vapor and CO_2_ movement, although without significantly affecting transpiration.

There were higher levels of iron and zinc in plant leaves in the industrial zone than the residential zone, which may have been due to emissions from the nearby steel and ceramic factories. Iron and zinc can be toxic to both humans and ecosystems [11]. Indeed, [57] reported that heavy metals can be absorbed directly from the air by leaves, depending on leaf adsorption capacity and physical characteristics as well as the plant species. In general, the heavy metals concentrations in water and soil were lower than allowed by local law (4/1994; 9/2009). Cadmium and copper were not detected in either zone, perhaps because they were not used as raw materials in any of the local industries. The U.S. EPA [41] regulates nine trace elements for land-applied sewage sludge (As, Cd, Cu, Pb, Hg, Mo, Ni, Se, and Zn), with six of these elements (Cu, Ni, Zn, Cd, Pb, and Se) considered to be phytotoxic [58]. Encouragingly, the quality of all groundwater samples was compatible with the Egyptian standards for drinking water.

Iron was the most abundant metal in soil in the industrial zone. Sulaiman and Hamzah [20] reported that heavy metal concentrations in roadside plants were higher than in the same species from uncontaminated sites. Metals in the root were only weakly transported to the stem but more strongly mobilized to leaves when available in the stems. Indeed, iron concentrations were higher in leaves of plants growing in the industrial zone, which is likely to have originated from the soil contaminated by nearby industrial activity and perhaps differences in traffic density between the different sites [59]. Lead, a toxic heavy metal, was also recorded in plants in the industrial zone but not in the residential zone. The high levels of Zn in the plants, water, and soil in the industrial zone compared to the residential zone could be attributable to metal-containing waste in the industrial zone, which might have leached into the underlying soil to be absorbed by plants [60]. Manganese was also recorded at high levels in plant leaves and soil in the industrial zone. Manganese is mainly derived from petroleum combustion and steel smelting. However, again, the concentrations of lead, manganese, and cadmium did not exceed the standard limits [61]. Nickel, detected at higher levels in plants leaves and soil in the industrial zone, is produced from the burning of coal and oil [62]. The toxicity and carcinogenicity of high doses of nickel are well documented and are caused by its potential to damage proteins and nucleic acids [63].

As well as being used as bioindicators, given the high uptake of pollutants in *B*. *glabra*, these plants may be useful for phytoremediation [64], which describes treating the environment with plant species which have a high capacity to accumulate pollutants. Finally, several studies have reported that some plant species (e.g., *Cupressus sempervirens*, *Pinus halepensis*) are more efficient at biomonitoring atmospheric pollutant [65, 66]. The ability of these plants to absorb and accumulate xenobiotics makes them useful as indicators of environmental pollution [67]. Further work is required to assess how *B*. *glabra* compares with other species for phytoremediation and bioindication.

## Conclusions

This study compared the presence and levels of pollutants between residential and industrial areas of a city and their possible effects on plants. Several types of pollutant were present at higher levels in *B*. *glabra* plants growing in industrial zones than those growing in residential zones. Furthermore, there were higher levels of phenolics and flavonoids in *B*. *glabra* leaves growing in industrial zones, which is likely to represent a stress response to pollutant exposure. Further work is necessary to establish whether *B*. *glabra* can be used for phytoremediation and as a useful bioindicator of environmental pollution.
